# Cost-effectiveness and budgetary impact of HCV treatment with direct-acting antivirals in India including the risk of reinfection

**DOI:** 10.1371/journal.pone.0217964

**Published:** 2019-06-06

**Authors:** Antoine Chaillon, Sanjay R. Mehta, Martin Hoenigl, Sunil S. Solomon, Peter Vickerman, Matthew Hickman, Britt Skaathun, Natasha K. Martin

**Affiliations:** 1 Division of Infectious Diseases and Global Public Health, Department of Medicine, University of California San Diego, La Jolla, California, United States of America; 2 Department of Medicine, San Diego Veterans Affairs Medical Center, La Jolla, California, United States of America; 3 Department of Medicine, Medical University of Graz, Graz, Austria; 4 Johns Hopkins University School of Medicine, Baltimore, Maryland, United States of America; 5 Population Health Sciences, University of Bristol, Bristol, United Kingdom; Centers for Disease Control and Prevention, UNITED STATES

## Abstract

**Background:**

HCV direct-acting antivirals (DAAs) are produced in India at low cost. However, concerns surrounding reinfection and budgetary impact limit treatment scale-up in India. We evaluate the cost-effectiveness and budgetary impact of HCV treatment in India, including reinfection.

**Methods:**

A closed cohort Markov model of HCV disease progression, treatment, and reinfection was parameterized. We compared treatment by fibrosis stage (F2-F4 or F0-F4) to no treatment from a health care payer perspective. Costs (2017 USD$, based on India-specific data) and health utilities (in quality-adjusted life years, QALYs) were attached to each health state. We assumed DAAs with 90% sustained viral response at $900/treatment and 1%/year reinfection, varied in the sensitivity analysis from 0.1–15%. We deemed the intervention cost-effective if the incremental cost-effectiveness ratio (ICER) fell below India’s per capita GDP ($1,709). We assessed the budgetary impact of treating all diagnosed individuals.

**Results:**

HCV treatment for diagnosed F2-F4 individuals was cost-saving (net costs -$2,881 and net QALYs 3.18/person treated; negative ICER) compared to no treatment. HCV treatment remained cost-saving with reinfection rates of 15%/year. Treating all diagnosed individuals was likely cost-effective compared to delay until F2 (mean ICER $1,586/QALY gained, 67% of simulations falling under the $1,709 threshold) with 1%/year reinfection. For all scenarios, annual retesting for reinfection was more cost-effective than the current policy (one-time retest). Treating all diagnosed individuals and reinfections results in net costs of $445–1,334 million over 5 years (<0.25% of total health care expenditure over 5 years), and cost-savings within 14 years.

**Conclusions:**

HCV treatment was highly cost-effective in India, despite reinfection. Annual retesting for reinfection was cost-effective, supporting a policy change towards more frequent retesting. A comprehensive HCV treatment scale-up plan is warranted in India.

## Introduction

An estimated 90% of the global burden of hepatitis C virus (HCV) falls within low to middle income countries (LMIC) [1]. India is one of the countries with the highest burden of HCV worldwide, with an estimated 6.1 million individuals chronically infected with HCV in 2016 [2, 3], roughly 8.5% of the global burden. Highly effective direct-acting antiviral treatments are now available, which are short duration (8–12 weeks), all-oral, highly tolerable, and can lead to cure in >90% of individuals. Yet in India and many LMICs, few are treated, despite advances in HCV treatment which have made HCV an easily curable infection [4]. Globally, it is estimated that only 7% of those diagnosed with HCV initiated treatment in 2015[5] and most LMIC settings have rates below these global estimates. Additionally, The World Health Organization (WHO) recently released a strategy to eliminate HCV as a public health threat, with targets to reduce HCV mortality by 65% and HCV incidence by 80% by 2030 [6]. Yet few LMIC have national strategies to reach this target [7].

India has been a global leader in producing generic HCV direct-acting antiviral therapies (DAAs) at a fraction of the cost compared to other countries, yet does not have a national HCV screening and treatment strategy to tackle the enormous burden of HCV. This is partially a result of ongoing concern about the potential risk of reinfection among the general population and budgetary impact of treatment (Vini Mahajan, Former Principal Secretary of Health and Family Welfare, Punjab, *personal comm)*. Also, unlike the National AIDS Control Organization that oversees HIV programming in India, there is currently no national organization targeted at elimination of viral hepatitis. A significant amount of HCV transmission in India is likely nosocomial, similar to other low- and middle-income country settings such as Pakistan and Egypt, but in contrast to many developed country settings where injection drug use is the predominant mode of transmission [8]. In India, HCV seroprevalence has been estimated to range from 0.4 to 1.9% [8–13]. Unsafe injection practices are common [14, 15], with a nation-wide population based cluster household survey finding that 27% reported receipt of injections, at an average rate of 2.9 injections/person/year–almost double that of Western Countries–and nearly half of these 3 billion injections are believed to be unsafe [16]. It has been estimated that 38% of HCV infections in India may be attributable to unsafe medical injections [17]. Individuals in India receive an average of 2.9 injections/person/year–almost double that of Western Countries–and nearly half of these 3 billion injections is believed to be unsafe [16]. Recent published work reported that HCV treatment with generic DAAs could be cost-saving in India for DAA costs up to $300 [18]. However, this analysis did not include the risk of reinfection, assess monitoring strategies post-treatment, or include setting-specific HCV-related disease management costs. Furthermore, no study has estimated the budgetary impact of treatment in India. In this study, we determine the cost-effectiveness and budgetary impact of HCV treatment and post-treatment monitoring with DAAs in India, including the potential risk of reinfection, utilizing India-specific HCV disease management costs.

## Method

### Overview

We performed a cost-effectiveness and budgetary impact analysis of HCV treatment provision among currently diagnosed individuals in India from a public sector health care payer perspective.

### Baseline and comparator

We compared the following scenarios:

1. No HCV treatment
2. Treatment of all HCV diagnosed individuals from F2-F4 with annual follow-up testing for reinfection and retreatment of reinfections
3. Treatment of all HCV diagnosed individuals from F0-F4 (i.e. universal HCV treatment) with annual follow-up testing for reinfection and retreatment of reinfections

### Model

We utilized a closed cohort Markov model of HCV disease progression, treatment, and reinfection among HCV diagnosed individuals (**S1 Fig**). The model tracked progression through HCV fibrosis stages (METAVIR F0/F1/F2/F3/F4[19]), decompensated cirrhosis (DC), hepatocellular carcinoma (HCC) and liver-related death. Liver transplantation was not considered in our model given general lack of availability of this intervention in the public sector [20]. For the purposes of this analysis, we assumed that all infected individuals without decompensated cirrhosis or cancer were eligible for treatment, although we note that in India individuals with decompensated cirrhosis are eligible for treatment, but would be selected on a case-by-case basis. We assumed that all individuals who achieve sustained viral response (SVR) with therapy are at risk of reinfection at a fixed rate per year. These individuals are retested annually, and re-infected individuals are eligible for retreatment. For our analysis, we assumed individuals whose treatment failed are ineligible for retreatment in the base-case. The model was stratified by genotype (genotype 3 vs. non-genotype 3) with liver disease progression rates accelerated among genotype 3 individuals.

### Cost-effectiveness methods

Cost (in 2017 USD $ and Indian Rupees [1 USD = 64.5 INR]) and health utilities (in quality-adjusted life years, QALYs) were attached to each health state. Costs and QALYs were discounted 3% per year in the base case scenario. This rate was chosen as it is the median value of discounted rates across different regions of India [21]. Due to uncertainty in underlying parameters, we performed a probabilistic uncertainty analysis where all epidemiological and disease transition probabilities, costs, and health benefits were randomly sampled from probabilistic distributions (**S1 Table**) to generate a total of 1,000 parameter sets. For each of the 1,000 parameter sets, the model was run and outputs generated. We ranked the interventions in terms of average total cost and calculated the mean incremental cost-effectiveness ratios (ICER, the mean change in total costs divided by the mean change in QALYs) for each intervention compared to its next least costly comparator. Based on WHO recommendations, we determined the intervention to be highly cost-effective if the ICER was below India’s per capita GDP ($1,709) compared to its next least costly comparator [22]. We additionally present results on the incremental costs and incremental QALY plane. For cost-effective interventions, we present the mean incremental costs and mean incremental QALYs for varying reinfection rates.

### Sensitivity analyses on cost-effectiveness results

Due to uncertainty in reinfection rates, we performed one-way sensitivity analyses where we varied reinfection rates from 0.1% to 15% per year, (see discussion in *Model parameterization* section). We also performed one-way sensitivity analyses to estimate the effects of changes in HCV drug related costs ($300 versus $900 USD at baseline), SVR (80%, 85% and 95% versus 90% at baseline), discount rate (0% for costs and health utilities and 6% for costs [the current Indian interest rate], versus 3% at baseline), time horizon (20 years versus 100 years from baseline), baseline fibrosis stage distribution (20% cirrhosis versus 13.4% at baseline), HCV treatment delivery costs (simplified on-treatment monitoring with two follow-up visits ($318) or removing the end of treatment RNA test as this test is recommended but optional ($378) versus $484 at baseline [**S2 Table**]), cheaper tests for confirmation of chronic HCV, such as HCV core Ag (estimated at $15 versus $108 USD for HCV viral load at baseline), reduced health care utilization for HCV related disease (50% accessing health care for HCV disease compared to 100% at baseline), and time-varying death rate by age instead of constant[23]. Finally, we examine the impact if individuals with SVR were only retested once (at 1 year post SVR), compared to annually for our baseline scenarios.

### Budgetary impact methods

We assessed the budgetary impact of treating all HCV-diagnosed individuals in 2018 plus their future reinfections over time horizons ranging from 5 to 20 years. We assessed the budgetary impact on undiscounted total treatment costs, total diagnostic costs (for monitoring of reinfection), and total HCV-related care costs of treating diagnosed infections and their associated diagnosed reinfections. As per our previous baseline analysis, we assumed a 1% per year reinfection rate.

### Model parameterization

All model parameters and references can be found in **S1 Table**.

### Baseline population characteristics

Our base case population included HCV chronically infected individuals aged 35 years at HCV diagnosis [24]. An estimated 6,151,257 individuals were chronically infected with HCV in India in 2016 [2, 3]. The numbers of HCV diagnosed individuals in India are uncertain, with estimates varying from 5% in 2013 [25], 6% in 2016 [3], up to an estimated 10% in 2017 based on discussions with country experts. For our analysis, we estimate that 10% of infections are diagnosed in 2017 as our base case scenario. The mean proportion of HCV infected individuals with HCV genotype 3 was set at 62%. The background mortality rate was estimated assuming a life expectancy at age 35 of 74 years. The HCV fibrosis distribution among HCV diagnosed individuals was: no fibrosis [F0]: 18.4%, portal fibrosis without septa [F1]: 24.8%, portal fibrosis with few septa [F2]: 21.7%, numerous septa without fibrosis [F3]: 21.7%, or cirrhosis [F4]:13.4% based on India-specific data [26].

### HCV reinfection rate

HCV reinfection rates are unknown in India. A systematic review and meta-analysis of HCV reinfection in the interferon-era found HCV reinfection rates of 0.185 per 100 person-years (/100py) (95% CI, 0.071–0.335/100py) among low risk individuals and 2.232/100py (95% CI, 1.307–3.346/100py) among high-risk individuals such as people who inject drugs and prisoners[27]. A recent large population-based cohort study in Canada with over 35,000 person-years of follow-up found reinfection rates of 1.27/100 person-years[28]. Unfortunately, these studies were from high-income countries without substantial community transmission. Primary incidence among the general population in Egypt, a setting with high HCV prevalence and ongoing community transmission, has been estimated at 0.55–0.74/100py [29, 30]. A modeling analyses in Pakistan, another setting with substantial community transmission estimated HCV primary incidence among non-PWID in the general population with high medical and community risks at 0.41–0.59/100py [31]. Due to risk heterogeneity, it is likely that reinfection rates in these settings are higher than these values, but there is a lack of empirical data. Given this uncertainty, we assumed a community reinfection rate of 1% per year, and examine the impact of varying reinfection rate from 0.1% to 15% per year in the sensitivity analyses.

### Disease stage transition probabilities

The estimates of stage-specific transition probabilities and mortality rates were obtained from published studies (see **S1 Table** for details). Rates of disease progression from F3 to cirrhosis and through ESLD were adjusted for HCV Genotype 3. Treated patients with F0-F3 fibrosis stages who achieved SVR were assumed to be cured and followed the general population mortality while individuals with cirrhosis or more advanced disease who achieved SVR could still progress at a reduced rate (see **S1 Table**).

### HCV treatment efficacy and costs

As limited data on the efficacy of new DAAs among populations in India are available, we assume a baseline DAA treatment efficacy (i.e. rate of SVR of 90% for all genotypes based on sofosbuvir/velpatasvir and other DAAs, which we varied in our sensitivity analyses. We assumed drug costs for DAAs of $900 per treatment, based on a 3 month course of sofosbuvir/velpatasvir as retailed in India [32] and the National Pharmaceutical Pricing Authority of India fixing the maximum retail price of 28 tablets of sofosbuvir/velpatasvir at INR 17,500 (USD 260) [33] in India including goods and service tax. We also evaluated reduced DAA costs ($300/treatment) in the sensitivity analysis. In addition to the drug costs, components of treatment delivery (pre-treatment and on-treatment monitoring) and associated costs were based upon the Metropolis Indian Directory of Services MetroEDOS [34, 35] and the latest Indian National Association for Study of the Liver (INASL) treatment guidelines[36]. In our baseline scenario, we assumed a total delivery cost per HCV treatment of $484 and varied +/-50% (see **S2 Table** for details).

### Disease stage costs

We utilized annual HCV disease related costs for F4/compensated cirrhosis (USD $538), DC ($4,353), and HCC ($5,698) based on published estimates from a public Central Government Health Scheme (CGHS) hospital [37]. As these costs are likely to vary across regions and hospitals, we sampled the costs uniformly ±50% from these estimates (**S1 Table)**.

### Utilities

Health utility data related to HCV in India are lacking, so health utilities (measured in quality adjusted life-years, QALYs) for each disease state (**S1 Table**) were sourced from previous studies from settings such as the United Kingdom (UK), consistent with previous economic evaluations for HBV-related interventions in India [38–41].

## Results

### Cost-effectiveness analysis

**Fig 1:**
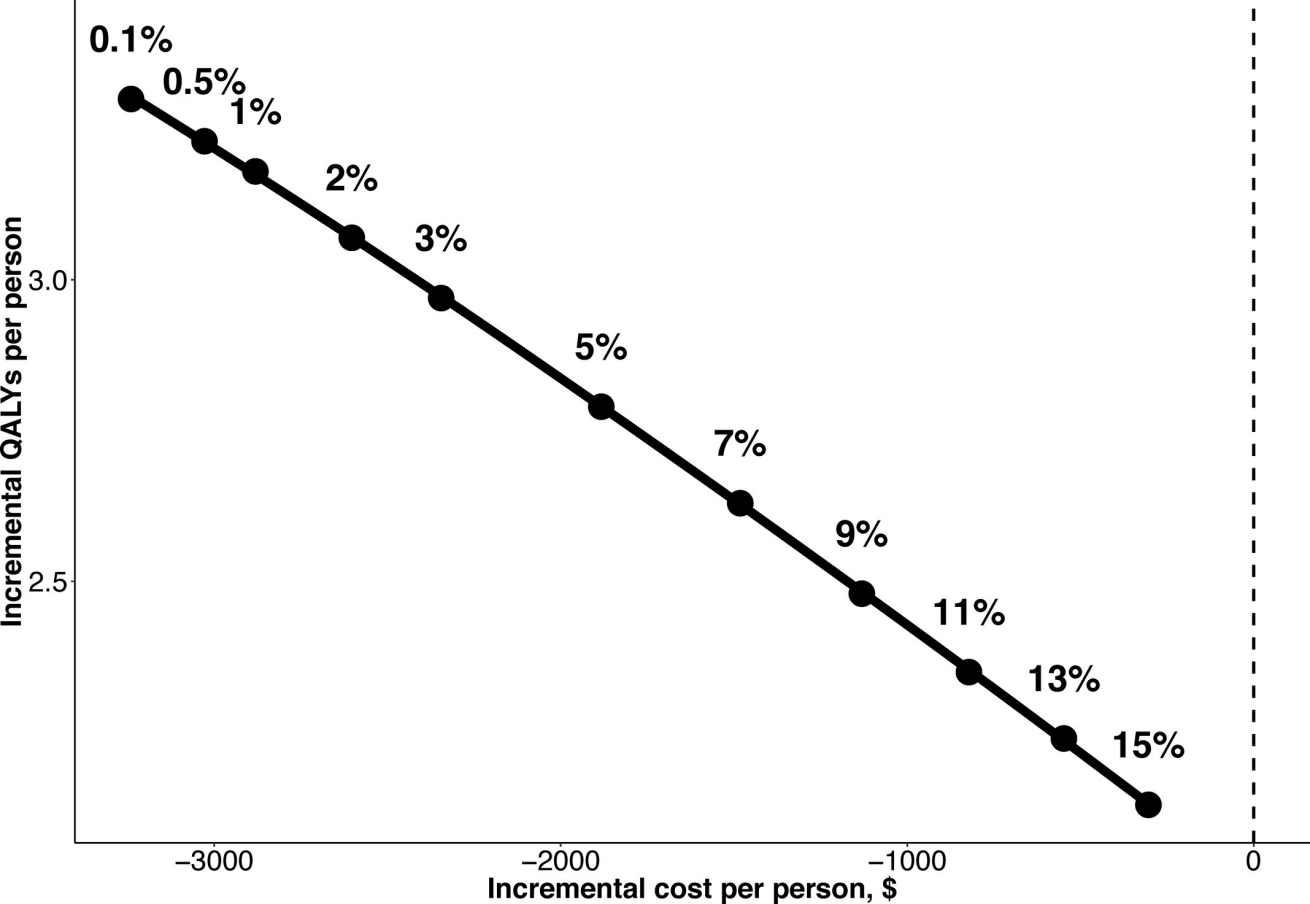
Incremental costs (horizontal axis) and QALYs (vertical axis) for treating F2-F4 fibrosis with DAAs in India compared to no treatment for various reinfection rates.

Using our baseline scenario of 1% reinfection per year, treating diagnosed individuals at moderate to severe liver disease stages (Fibrosis F2-F4) was cost-saving compared to no treatment (net costs -$2,881/person treated and net QALYs 3.18/person treated; **Table 1**). Treating all diagnosed individuals with moderate-severe liver disease remained cost-saving compared to no treatment with all reinfection rates evaluated (up to 15% per year, **Fig 1**).

**Table 1:** Sensitivity analyses of cost-effectiveness results for HCV treatment for individuals with moderate-severe liver disease (F2-F4) versus no treatment. ICER: Incremental cost-effectiveness ratio. QALY: quality adjusted life year. SVR: Sustained viral response.

Values of parameter being varied	Mean Costs* per person		Mean QALYs per person		Mean Incremental Costs*	Mean Incremental QALYs	Mean ICER ($/QALY gained)
	No Treatment	Treat F2-F4.	No Treatment	Treat F2-F4			
Base-Case	8087	5206	10.78	13.96	-2881	3.18	Cost Saving
Drug Costs							
$300	8087	4676	10.78	13.96	-3411	3.18	Cost Saving
SVR Rate							
80%	8087	5681	10.78	13.57	-2405	2.79	Cost Saving
85%	8087	5445	10.78	13.77	-2642	2.98	Cost Saving
95%	8087	4963	10.78	14.15	-3124	3.37	Cost Saving
Discount Rate							
0% cost and 0% QALYs	15558	9486	16.4	26.67	-6072	10.27	Cost Saving
6% cost and 3% QALYs	4994	3644	10.78	13.96	-1350	3.18	Cost Saving
Fibrosis Stage distribution							
F0: 17.3%, F1: 23.3%, F2: 19.7%, F3: 19.7%, F4: 20%	8652	5956	10.51	13.73	-2696	3.21	Cost Saving
Laboratory and Visit Cost							
$318	8087	5056	10.78	13.96	-3031	3.18	Cost Saving
Without final RNA ($378)	8087	5035	10.78	13.96	-3617	3.18	Cost Saving
Disease Stage Cost							
50% decrease	4043	3810	10.78	13.96	-233	3.18	Cost Saving
Time horizon							
20 years	4943	3713	8.73	9.69	-1230	0.95	Cost Saving
Screening Periodicity							
One-time screening	8087	5899	10.78	13.61	-2187	2.83	Cost Saving
Confirmation Method							
Core Antigen (unit Cost = $)	8087	4192	10.78	13.96	-3895	3.18	Cost Saving
Background Mortality							
Time-varying death rate	10681	6211	13.3	17.02	-4470	3.76	Cost Saving

*In 2017 USD$.

**Fig 2:**
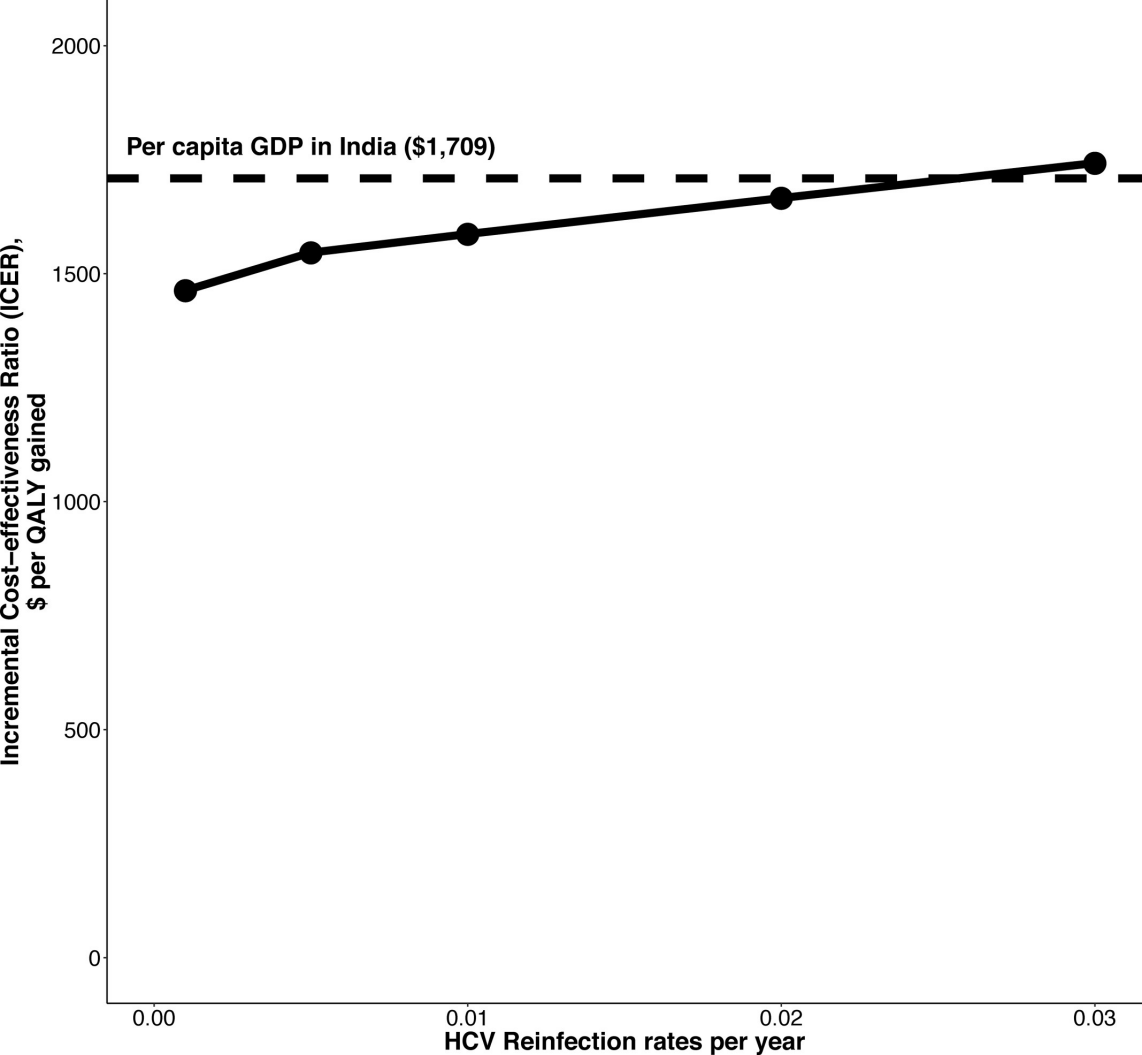
Incremental cost-effectiveness ratio of treating F0-F4 compared to F2-F4 in India for various reinfection rates from 0.5–3% per year. Dashed line shows the ‘highly cost-effective’ 1xGDP threshold for India ($1,709). ICER: Incremental cost-effectiveness ratio.

Treating all diagnosed individuals (including people with mild disease, F0-F4) was more costly than treatment for only those with moderate-severe liver disease (F2-F4) but resulted in greater benefits. At a 1% reinfection rate, treating all diagnosed individuals was likely cost-effective compared to treating those with moderate-severe liver disease, with a mean ICER of $1,586/QALY gained (**Table 1**). In this scenario, 67% of the simulations produced ICERs below the 1-times per capita GDP cost-effectiveness threshold for India ($1,709/QALY gained). Treating all diagnosed individuals remained cost-effective compared to treating those with moderate-severe liver disease with reinfection rates below 3% (**Fig 2**).

Our base case results with 1% reinfection/year were robust to changes in several parameters. **Table 1** shows the sensitivity analyses on the cost-effectiveness results of treatment for those with moderate-severe liver disease (F2-F4) compared to no treatment. If follow-up testing for reinfection is less frequent (once at 1 year after SVR compared to annually), treatment remains cost-saving but fewer costs are saved as many reinfections are left undiagnosed and untreated. HCV treatment for individuals with moderate-severe liver disease remained cost-saving compared to no treatment for all other sensitivity analyses examined, including a lower SVR rate of 85% (vs 90% at baseline), higher discount rate for costs (6% vs 3% at baseline), higher proportion of cirrhosis at the initial stage distribution (20% vs 13.4% at baseline). **Table 2** shows the sensitivity analyses on our results evaluating treatment of all diagnosed (F0-F4) compared to those with moderate-severe liver disease (F2-F4). HCV treatment for all diagnosed remained cost-effective (ICER<$1,709) for SVR rates as low as 80%, and for scenarios examining different discount rates, cirrhosis distributions, and treatment costs. However, this scenario did not remain cost-effective with a strategy of only one-retest for reinfection at one year or with a time horizon of 20 years (ICER $2,755).

**Table 2:** Sensitivity analyses of cost-effectiveness results treating all diagnosed individuals (F0-F4 fibrosis) compared to targeting those with moderate-severe liver disease (F2-F4). ICER: Incremental cost-effectiveness ratio. QALY: quality adjusted life year. SVR: Sustained viral response.

Values of parameter being varied	Mean Costs* per person		Mean QALYs per person		Mean Incremental Cost*	Mean Incremental QALYs	Mean ICER ($/QALY gained)
	Treating F2-F4	Treating F0-F4	Treating F2-F4	Treating F0-F4			
Base case	5206	5857	13.96	14.37	651	0.41	1586
Drug Costs							
$300	4676	5170	13.96	14.37	495	0.41	1204.7
SVR Rate							
80%	5681	6294	13.57	13.94	613	0.36	1700.2
85%	5445	6077	13.77	14.15	632	0.39	1640.4
95%	4963	5634	14.15	14.59	671	0.44	1538.4
Discount Rate							
0% cost and 0% QALYs	9486	10084	26.67	27.58	598	0.91	658.9
6% cost and 3% QALYs	3644	4291	13.96	14.37	647	0.41	1576.2
Reinfection Rate							
0.1%	4847	5481	13.93	14.37	634	0.43	**1463.1**
0.5%	5059	5703	14.01	14.43	644	0.42	**1546.1**
2%	5484	6149	13.85	14.25	665	0.4	**1665.8**
3%	5742	6420	13.75	14.14	679	0.39	1742.5
5%	6204	6907	13.57	13.94	703	0.37	1892.1
7%	6605	7332	13.41	13.77	727	0.36	2039.1
9%	6956	7705	13.26	13.61	749	0.34	2185.4
11%	7265	8036	13.14	13.47	771	0.33	2331.5
13%	7539	8331	13.02	13.34	792	0.32	2477.8
15%	7783	8595	12.92	13.23	812	0.31	2624.1
Fibrosis Stage Distribution							
F0: 17.3%, F1: 23.3%, F2: 19.7%, F3: 19.7%, F4: 20%	5956	6569	13.73	14.11	612	0.39	1586.9
Laboratory and Visit Cost							
$318	5056	5663	13.96	14.37	607	0.41	1478.4
Without 3^rd^ RNA ($378)	5034	5654	13.96	14.37	619	0.41	1507
Disease Stage Cost							
50% decrease	3810	4482	13.96	14.37	672	0.41	1636.1
Time horizon							
20 years	3713	4391	9.69	9.9	677	0.25	2754.5
Screening Periodicity							
One-time screening	8087	6850	13.61	13.97	951	0.36	2639.9
Confirmation Method							
Core Antigen (unit Cost = $)	4192	4563	13.96	14.37	371	0.41	903.3
Background Mortality							
Time-varying death rate	6211	6879	17.02	17.55	668	0.53	1256

*In 2017 USD$.

### Budgetary impact

**Fig 3:**
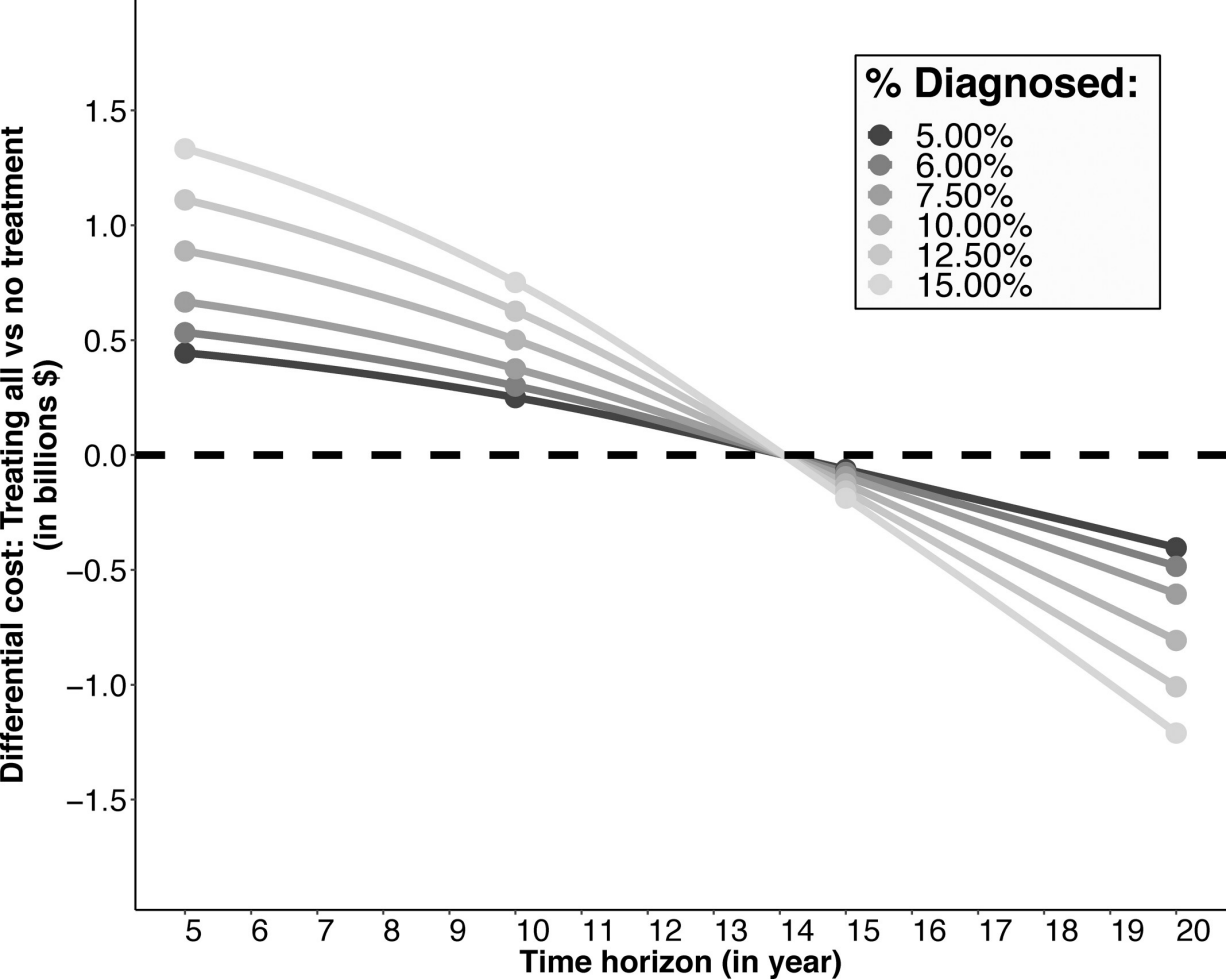
Budgetary impact (shown in differential costs) of treating all diagnosed individuals compared to no treatment over various time horizons from 5–20 years and percentage diagnosed from 5 to 15% of the chronically infected population.

If all diagnosed individuals in 2018 are treated plus their future reinfections (an estimated 615,126 individuals) this would require approximately $1.48 billion in treatment and reinfection monitoring costs during the next 5 years, but save $211 million in costs related to HCV disease, resulting in a net cost of $887 million compared to no treatment over 5 years (**Table 3**). Estimates are highly sensitive to assumptions regarding the proportion of the HCV-infected population who are diagnosed. If 5% of HCV-infected individuals are diagnosed in 2018, treating all diagnosed would result in a net cost of $445 million compared to no treatment over 5 years, whereas if 15% of HCV-infected individuals are diagnosed this results in a net cost of $1.33 billion (**Fig 3**). Total health expenditure in India is an estimated 3.9% of GDP [42], which equates to roughly $110 billion in 2018. Hence, the net cost of treating all diagnosed individuals would cost less than 0.25% of total health expenditure over 5 years. However, treating all diagnosed individuals would become cost-saving within 14 years, regardless of the number of diagnosed individuals (**Fig 3**).

**Table 3:** Budgetary impact of treating all HCV diagnosed individuals in 2018 for various time horizons, assuming 10% of the HCV infected population is diagnosed.

Time horizon		Number of infections treated/ retreated Mean (2.5–97.5% interval)	Total Costs (million USD$)	Total HCV disease care costs (million USD$)	Total HCV treatment cost (million USD$)	Reinfection test costs (million USD$)	Differential Cost (million USD$)
5 years	No treatment	0	596.7 (368.3–839.9)	596.7 (368.3–839.9)	0	0	
	Treat all diagnosed in 2018 and associated reinfections	650,801 (647,215–653,655)	1483.3 (1255.5–1709)	385.8 (241.2–539.3)	906.6 (751.6–1052.1)	190.8 (188.9–192.2)	886.6 (677.6–1067.4)
10 years	No treatment	0	1663.1 (1039.8–2328.6)	1663.1 (1039.8–2328.6)	0	0	
	Treat all diagnosed in 2018 and associated reinfections	674,492 (670,311–677,857)	2144.2 (1804.8–2487.2)	778.7 (495.3–1085.3)	939.6 (779–1090.8)	425.9 (420.0–430.3)	481.1 (-2-881.8)
15 years	No treatment	0	2902.7 (1814.5–4084.3)	2902.7 (1814.5–4084.3)	0	0	
	Treat all diagnosed in 2018 and associated reinfections	692,905 (688,152–696,658)	2730.9 (2278.9–3195)	1135.2 (728.6–1570.1)	965.3 (800.3–1120.9)	630.5 (619.9–638.4)	-171.8 (-1046.6–542.7)
20 years	No treatment	0	4135.1 (2615.5–5895.7)	4135.1 (2615.5–5895.7)	0	0	
	Treat all diagnosed in 2018 and associated reinfections	708,909 (703,588–713,082)	3250.5 (2692.7–3832.5)	1454.8 (936.7–2009.2)	987.5 (818.4–1146.8)	808.2 (792.6–819.9)	-884.6 (-2196.6–164.3)

## Discussion

Our analysis highlights that despite the risk of reinfection among the general population in India, HCV treatment for diagnosed individuals with moderate to severe liver disease (fibrosis F2 or greater) and annual monitoring for reinfection in India is cost-saving compared to no treatment. Additionally, a further expansion to treat all diagnosed (F0 or above) is likely cost-effective compared to treating those with moderate to severe liver disease. Together these results support the provision of HCV treatment among the general population in India, despite any potential additional associated costs related to retesting and retreatment due to reinfection. Our analysis additionally found that annual retesting for reinfection among those treated for HCV was more cost-effective than a onetime retest after sustained viral response. As current Indian guidelines only recommend a one-time retest at one year post SVR, our work supports a policy change towards annual retesting [36, 43]. Additionally, although we found that treating all diagnosed individuals in 2018 and their future reinfections would incur substantial costs to the government, at a net cost of $445–1,334 million over the next 5 years (<0.25% of total health expenditure over 5 years), it would become cost-saving within 14 years. As HCV is a slowly progressing disease, the economic benefits to the health payer occur over a longer time frame, but nevertheless can result in overall cost-savings within a moderate time frame.

### Strengths and weaknesses

To our knowledge, we present the first cost-effectiveness study of HCV treatment including the risk of reinfection in India, and the first estimates of budgetary impact of a HCV treatment strategy in India. Our study additionally builds on previous work to incorporate published India-specific costs related to management of untreated HCV disease.

Our study has several important limitations, mainly due to parameter uncertainty. First, the reinfection rate among individuals infected with HCV in India is unknown, so we provide extensive sensitivity analyses varying from 0.1% to 15% per year. Our sensitivity analyses show our main conclusions are robust to reinfection rates up to 3%/year, which approach rates found in PWID populations and are likely higher than those with community risks which comprise the majority of the HCV-infected population in India[27]. If, on the other hand, reinfection rates are lower than 1%, our analysis shows that HCV treatment is even more cost-effective, as fewer require retreatment. Indeed, it is possible that the experience of undergoing HCV treatment reduces an individual’s risk behavior, although data are lacking on this, particularly in the DAA era. Nevertheless, our findings that HCV treatment is cost-effective are robust to lower reinfection rates than our baseline.

Second, there is substantial uncertainty in other parameters such as health resource utilization among individuals chronically infected with HCV and the proportion of the chronically infected population who are diagnosed. Nevertheless, we found that our cost-effectiveness results were robust to sensitivity analyses examining a case where individuals present to the hospital less often and therefore accrue fewer costs associated to untreated HCV. Additionally, we present the expected budgetary impact given a wide range of diagnosis rates, and note that our results indicate that HCV treatment becomes cost-saving at 14 years regardless of diagnosis prevalence.

Third, our analysis utilizes a fixed rate of reinfection and therefore does not incorporate any potential prevention benefits on transmission for treating those who retain an ongoing risk of transmission/reinfection. Other studies of HCV treatment for people at ongoing risk of transmission (such as people who inject drugs) have found that targeting these populations can be more cost-effective than treating those with no ongoing risk due to the substantial prevention benefits. As such, HCV treatment could be even more cost-effective than we propose.

Fourth, our analysis focuses solely on the cost-effectiveness and budgetary impact of treatment of diagnosed individuals, and as such does not examine HCV screening. Given that the vast majority of HCV-infected individuals in India are undiagnosed, an effective HCV elimination strategy will need to incorporate both HCV screening and treatment components. Further work should examine the cost-effectiveness of HCV screening strategies in India.

Finally, our estimates of budgetary impact are sensitive to DAA cost, and likely overestimates if the government is able to procure HCV treatments and diagnostics at lower prices. For example, if treatments can be obtained at prices similar to what was negotiated in the state of Punjab (estimated around $100 per 12 week treatment course and $30 per HCV RNA) [44], then it would reduce the budgetary impact and shorten the time for treatment to be cost-saving. Additionally, it is possible that HCV treatment algorithms will be streamlined in the future, with reduced on-treatment monitoring and potentially elimination of end-of treatment viral load, which could additionally reduce costs.

### Comparison with other published studies

Despite a wide body of literature examining the cost-effectiveness of HCV treatment in high-income settings [45–48], few studies have examined the cost-effectiveness of HCV therapy in low or middle income countries. Our results are consistent with a previous publication showing that generic DAA therapy in India for those not at risk of reinfection and regardless of fibrosis stage is cost-effective compared to no treatment at a treatment cost of $900, and cost-saving at a drug cost of $100 [18]. Together these studies strongly support the cost-effectiveness of HCV treatment among the general population in India. Our results are also consistent with studies in high-income country settings that show that HCV treatment with DAAs among people at risk of reinfection such as PWID is cost-effective, despite the risk of reinfection [49, 50].

## Conclusion

The enormous burden of HCV in India and the availability of highly effective and affordable HCV DAA therapies in India poses an enormous opportunity. HCV treatment and annual monitoring for reinfection among the general population in India is likely cost-effective and potentially cost-saving, despite the risk of reinfection. We found that annual retesting for reinfection among those treated for HCV was more cost-effective than a onetime retest after sustained viral response, supporting a policy change in India towards more frequent retesting. Although HCV treatment for all diagnosed individuals would result in a large budgetary impact, this investment would be offset within roughly 15 years due to averted HCV related health care costs. This evidence points towards the benefit and need for a comprehensive HCV treatment action plan in India.

## Supporting information

S1 Table Model parameters and references. (DOCX)

S2 Table HCV treatment delivery costs and visits based on Indian treatment guidelines and India-specific prices, translated to 2017 USD. *Extensive liver tests (ie, albumin, total and direct bilirubin, alanine aminotransferase [ALT], aspartate aminotransferase [AST], and alkaline phosphatase levels)
(DOCX)

S1 Fig Schematic of the HCV natural history and treatment model disease stages (boxes) and transitions (arrows). F0, F1, F2, F3, F4 denote METAVIR stages. SVR: sustained viral response. DC: decompensated cirrhosis. HCC: hepatocellular carcinoma. We assume no liver transplant and individuals diagnosed with DC/HCC are not treated for HCV. Individuals who failed treatment are not re-treated.
(DOCX)
