# Supplementary material for: Cost-effectiveness and budgetary impact of HCV treatment with direct-acting antivirals in India including the risk of reinfection
Source: PLoS One. 2019 Jun 6;14(6):e0217964. doi: 10.1371/journal.pone.0217964 (PMC6553784; doi:10.1371/journal.pone.0217964)
Supplement: S1 Table — (DOCX) [file pone.0217964.s001.docx]

**S1 Table. Model parameters and references.**

| **State Transitions** | **HCV Stage** | **Mean sampled value (2.5%-97.5% quantiles)** | **Sampling distribution** | **Source** |
| --- | --- | --- | --- | --- |
| Annual probability of a liver disease stage transition | F0 to F1 | 0.117 (0.105-0.129) | Uniform (min-max 0.104-0.130) | [1] |
|  | F1 to F2 | 0.085 (0.076-0.095). | Uniform (min-max 0.075-0.096). | [1] |
|  | F2 to F3 | 0.120 (0.110-0.132) | Uniform (min-max 0.109-0.133) | [1] |
|  | F3 to F4 | 0.116 (0.104-0.128) | Uniform (min-max 0.104-0.129) | [1] |
|  | F4 to DC | 0.039 (0.030-0.081) | Beta (14.617,260.1732) | [2] |
|  | F4 to HCC | 0.014 (0.016-0.038) | Beta (1.9326,136.1074) | [2] |
|  | DC to HCC | 0.014 (0.002-0.037) | Beta (1.9326,136.1074) | [2] |
| SVR Probability |  | 0.9 |  | [3-5] |
| Proportion Genotype 3 |  | 0.618 (0.598-0.638) | Uniform (min-max: 0.597-0.639) | [6-11] |
| Annual discount rate of costs and QALYs |  | 0.03 |  | [12] |
| Relative risk of Disease progression if infected by Genotype 3 | F3 to F4 | 1.31 (1.223-1.386) | lognormal | [13, 14] |
|  | F4 to DC | 1.31 (1.223-1.386) | lognormal |  |
|  | F4 and DC to HCC | 1.8 (1.621-2.018) | lognormal |  |
| Annual probability of liver-related death while in a given ESLD compartment | F4 | 0 |  |  |
|  | DC | 0.13 (0.111-0.151) | Beta (147,984) | [2, 15, 16] |
|  | HCC | 0.43 (0.372-0.487) | Beta (117.1, 155.2) |  |
| Annual probability of background mortality assuming a baseline age at entry of 35 years | All Stage | 0.0256 |  | [17, 18] |
| Relative risk of progression from F4 to DC if SVR | F4 to DC | 0.07 (0.023-0.187) | lognormal | [19] |
| Relative risk of progression from F4 to HCC if SVR | F4 to HCC | 0.23 (0.148-0.345) | lognormal | [19, 20] |
| Relative risk of progression from DC to HCC if SVR | DC to HCC | 1 | - |  |
| **Cost (US$)** |  |  |  |  |
| Annual costs for non-treatment medical expenses among HCV-infected patients | F0, F1, F2 | 0 | - |  |
|  | F3, F4 | 546 (283-794) | Uniform (min-max 269-807) | [21] |
|  | DC | 4321 (2302-6414) | Uniform (min-max 2176- 6530) | [21] |
|  | HCC | 5668 (3004-8364) | Uniform (min-max 2849-8547) | [21] |
| HCV antiviral therapy per treatment |  | 900 |  | [22] |
| Treatment Delivery Cost (see S2 Table for details) |  | 486 (257-720) | Uniform (min-max 243-729) | [23-25] |
| **Health Utilities** |  |  |  |  |
| HCV-infected patients | F0, F1 | 0.848 (0.738-0.963) | Uniform (min-max: 0.73-0.97) | [26-28] |
|  | F2, F3 | 0.766 (0.666-0.873) | Uniform (min-max: 0.66-0.88) |  |
|  | F4 | 0.73 (0.582-0.881) | Uniform (min-max: 0.57-0.89) |  |
|  | DC | 0.602 (0.458-0.742) | Uniform (min-max: 0.45-0.75) |  |
|  | HCC | 0.375 (0.102-0.651) | Uniform (min-max: 0.09-0.67) |  |
| Incremental increase in health utility upon SVR |  | 0.05 |  | [29] |

**REFERENCES**

1. Thein HH, Yi Q, Dore GJ, Krahn MD. Natural history of hepatitis C virus infection in HIV-infected individuals and the impact of HIV in the era of highly active antiretroviral therapy: a meta-analysis. Aids. 2008;22(15):1979-91. Epub 2008/09/12. doi: 10.1097/QAD.0b013e32830e6d51. PubMed PMID: 18784461.

2. Shepherd J, Jones J, Hartwell D, Davidson P, Price A, Waugh N. Interferon alpha (pegylated and non-pegylated) and ribavirin for the treatment of mild chronic hepatitis C: a systematic review and economic evaluation. Health technology assessment (Winchester, England). 2007;11(11):1-205, iii. Epub 2007/03/10. PubMed PMID: 17346498.

3. Ampuero J, Reddy KR, Romero-Gomez M. Hepatitis C virus genotype 3: Meta-analysis on sustained virologic response rates with currently available treatment options. World journal of gastroenterology. 2016;22(22):5285-92. doi: 10.3748/wjg.v22.i22.5285. PubMed PMID: PMC4893476.

4. Organization WH. Guidelines for the Screening Care and Treatment of Persons with Chronic Hepatitis C Infection: Updated Version. 2016.

5. EASL Recommendations on Treatment of Hepatitis C 2016. J Hepatol. 2017;66(1):153-94. Epub 2016/09/27. doi: 10.1016/j.jhep.2016.09.001. PubMed PMID: 27667367.

6. Christdas J, Sivakumar J, David J, Daniel HD, Raghuraman S, Abraham P. Genotypes of hepatitis C virus in the Indian sub-continent: a decade-long experience from a tertiary care hospital in South India. Indian journal of medical microbiology. 2013;31(4):349-53. Epub 2013/09/26. doi: 10.4103/0255-0857.118875. PubMed PMID: 24064640.

7. Raghuraman S, Shaji RV, Sridharan G, Radhakrishnan S, Chandy G, Ramakrishna BS, et al. Distribution of the different genotypes of HCV among patients attending a tertiary care hospital in south India. Journal of clinical virology : the official publication of the Pan American Society for Clinical Virology. 2003;26(1):61-9. Epub 2003/02/19. PubMed PMID: 12589835.

8. Chakravarti A, Dogra G, Verma V, Srivastava AP. Distribution pattern of HCV genotypes & its association with viral load. Indian J Med Res. 2011;133:326-31. Epub 2011/03/29. PubMed PMID: 21441689; PubMed Central PMCID: PMCPMC3103160.

9. Narahari S, Juwle A, Basak S, Saranath D. Prevalence and geographic distribution of Hepatitis C Virus genotypes in Indian patient cohort. Infection, genetics and evolution : journal of molecular epidemiology and evolutionary genetics in infectious diseases. 2009;9(4):643-5. Epub 2009/05/23. doi: 10.1016/j.meegid.2009.04.001. PubMed PMID: 19460332.

10. Verma V, Chakravarti A, Kar P. Genotypic characterization of hepatitis C virus and its significance in patients with chronic liver disease from Northern India. Diagnostic microbiology and infectious disease. 2008;61(4):408-14. Epub 2008/05/06. doi: 10.1016/j.diagmicrobio.2008.03.011. PubMed PMID: 18455899.

11. Messina JP, Humphreys I, Flaxman A, Brown A, Cooke GS, Pybus OG, et al. Global distribution and prevalence of hepatitis C virus genotypes. Hepatology. 2015;61(1):77-87. Epub 2014/07/30. doi: 10.1002/hep.27259. PubMed PMID: 25069599; PubMed Central PMCID: PMCPMC4303918.

12. Gold MR, Franks P, McCoy KI, Fryback DG. Toward consistency in cost-utility analyses: using national measures to create condition-specific values. Med Care. 1998;36(6):778-92. Epub 1998/06/18. PubMed PMID: 9630120.

13. Kanwal F, Kramer JR, Ilyas J, Duan Z, El-Serag HB. HCV genotype 3 is associated with an increased risk of cirrhosis and hepatocellular cancer in a national sample of U.S. Veterans with HCV. Hepatology. 2014;60(1):98-105. Epub 2014/03/13. doi: 10.1002/hep.27095. PubMed PMID: 24615981; PubMed Central PMCID: PMCPMC4689301.

14. Probst A, Dang T, Bochud M, Egger M, Negro F, Bochud PY. Role of hepatitis C virus genotype 3 in liver fibrosis progression--a systematic review and meta-analysis. J Viral Hepat. 2011;18(11):745-59. Epub 2011/10/14. doi: 10.1111/j.1365-2893.2011.01481.x. PubMed PMID: 21992794.

15. Grieve R, Roberts J, Wright M, Sweeting M, DeAngelis D, Rosenberg W, et al. Cost effectiveness of interferon alpha or peginterferon alpha with ribavirin for histologically mild chronic hepatitis C. Gut. 2006;55(9):1332-8. Epub 2005/07/05. doi: 10.1136/gut.2005.064774. PubMed PMID: 15994216; PubMed Central PMCID: PMCPMC1860032.

16. Wright M, Goldin R, Fabre A, Lloyd J, Thomas H, Trepo C, et al. Measurement and determinants of the natural history of liver fibrosis in hepatitis C virus infection: a cross sectional and longitudinal study. Gut. 2003;52(4):574-9. Epub 2003/03/13. PubMed PMID: 12631672; PubMed Central PMCID: PMCPMC1773621.

17. Chowdhury A, Santra A, Chaudhuri S, Dhali GK, Chaudhuri S, Maity SG, et al. Hepatitis C virus infection in the general population: a community-based study in West Bengal, India. Hepatology. 2003;37(4):802-9. Epub 2003/04/02. doi: 10.1053/jhep.2003.50157. PubMed PMID: 12668973.

18. World Health Organization W. Global Health Observatory data repository 2017 [06/17/2017]. Available from: <http://apps.who.int/gho/data/>.

19. van der Meer AJ, Veldt BJ, Feld JJ, Wedemeyer H, Dufour JF, Lammert F, et al. Association between sustained virological response and all-cause mortality among patients with chronic hepatitis C and advanced hepatic fibrosis. Jama. 2012;308(24):2584-93. Epub 2012/12/27. doi: 10.1001/jama.2012.144878. PubMed PMID: 23268517.

20. Morgan RL, Baack B, Smith BD, Yartel A, Pitasi M, Falck-Ytter Y. Eradication of hepatitis C virus infection and the development of hepatocellular carcinoma: a meta-analysis of observational studies. Ann Intern Med. 2013;158(5 Pt 1):329-37. Epub 2013/03/06. doi: 10.7326/0003-4819-158-5-201303050-00005. PubMed PMID: 23460056.

21. Marfatia S, Gupta K, Mukherjee A, Mattoo V. Direct Medical Cost Associated With The Diagnosis and Treatment of Patients With Chronic Hepatitis-B In Three Large Metropolitan Cities In India - A Pilot Study. Value in health : the journal of the International Society for Pharmacoeconomics and Outcomes Research. 2015;18(7):A581-2. Epub 2015/11/05. doi: 10.1016/j.jval.2015.09.1945. PubMed PMID: 26533268.

22. Services MHLIDo. 2017 [cited 2017 11/03/2017]. Available from: [http://edos.metropolisindia.com/ - /MetroEDOS](http://edos.metropolisindia.com/#/MetroEDOS).

23. Metropolis Laboratory 2018 [cited 2018 01/23/2018]. Available from: <http://www.metropolisindia.com/gastromet/test-menu/>.

24. AASLD-IDSA. Recommendations for testing, managing, and treating hepatitis C.

25. Puri P, Saraswat VA, Dhiman RK, Anand AC, Acharya SK, Singh SP, et al. Indian National Association for Study of the Liver (INASL) Guidance for Antiviral Therapy Against HCV Infection: Update 2016. Journal of clinical and experimental hepatology. 2016;6(2):119-45. Epub 2016/08/06. doi: 10.1016/j.jceh.2016.07.001. PubMed PMID: 27493460; PubMed Central PMCID: PMCPMC4963318.

26. Leidner AJ, Chesson HW, Xu F, Ward JW, Spradling PR, Holmberg SD. Cost-effectiveness of hepatitis C treatment for patients in early stages of liver disease. Hepatology. 2015;61(6):1860-9. Epub 2015/02/14. doi: 10.1002/hep.27736. PubMed PMID: 25677072.

27. Townsend R, McEwan P, Kim R, Yuan Y. Structural frameworks and key model parameters in cost-effectiveness analyses for current and future treatments of chronic hepatitis C. Value in health : the journal of the International Society for Pharmacoeconomics and Outcomes Research. 2011;14(8):1068-77. Epub 2011/12/14. doi: 10.1016/j.jval.2011.06.006. PubMed PMID: 22152176.

28. Deuffic-Burban S, Schwarzinger M, Obach D, Mallet V, Pol S, Pageaux GP, et al. Should we await IFN-free regimens to treat HCV genotype 1 treatment-naive patients? A cost-effectiveness analysis (ANRS 95141). J Hepatol. 2014;61(1):7-14. Epub 2014/03/22. doi: 10.1016/j.jhep.2014.03.011. PubMed PMID: 24650691.

29. Wright M, Grieve R, Roberts J, Main J, Thomas HC. Health benefits of antiviral therapy for mild chronic hepatitis C: randomised controlled trial and economic evaluation. Health technology assessment (Winchester, England). 2006;10(21):1-113, iii. Epub 2006/06/06. PubMed PMID: 16750059.
