# Supplementary material for: Cost-effectiveness and budgetary impact of HCV treatment with direct-acting antivirals in India including the risk of reinfection
Source: PLoS One. 2019 Jun 6;14(6):e0217964. doi: 10.1371/journal.pone.0217964 (PMC6553784; doi:10.1371/journal.pone.0217964)
Supplement: S2 Table — *Extensive liver tests (ie, albumin, total and direct bilirubin, alanine aminotransferase [ALT], aspartate aminotransferase [AST], and alkaline phosphatase levels) (DOCX) [file pone.0217964.s002.docx]

**S2 Table. HCV treatment delivery costs and visits based on Indian treatment guidelines and India-specific prices, translated to 2017 USD.**  *Extensive liver tests (ie, albumin, total and direct bilirubin, alanine aminotransferase [ALT], aspartate aminotransferase [AST], and alkaline phosphatase levels)

|  | **Week 0** | **Weeks 4** | **Week 8** | **Week 12** | **SVR 12** |
| --- | --- | --- | --- | --- | --- |
| HCV RNA | $107.70 | - | - | $107.70 | $107.70 |
| Blood Count | $2.90 | $2.90 | $2.90 | $2.90 | $2.90 |
| Liver tests | $21.50* | $6.90 | $6.90 | $6.90 | $6.90 |
| Outpatient Visit | $20 | $20 | $20 | $20 | $20 |
| **Total** | **$152** | **$29** | **$29** | **$137** | **$137** |
