# Supplementary material for: Cost-effectiveness and budgetary impact of HCV treatment with direct-acting antivirals in India including the risk of reinfection
Source: PLoS One. 2019 Jun 6;14(6):e0217964. doi: 10.1371/journal.pone.0217964 (PMC6553784; doi:10.1371/journal.pone.0217964)
Supplement: S1 Fig — F0, F1, F2, F3, F4 denote METAVIR stages. SVR: sustained viral response. DC: decompensated cirrhosis. HCC: hepatocellular carcinoma. We assume no liver transplant and individuals diagnosed with DC/HCC are not treated for HCV. Individuals who failed treatment are not re-treated. (DOCX) [file pone.0217964.s003.docx]

**S1 Figure. Schematic of the HCV natural history and treatment model disease stages (boxes) and transitions (arrows).** SVR: sustained viral response. HCC: hepatocellular carcinoma. We assume no liver transplant and individuals diagnosed with DC/HCC are not treated for HCV. Individuals who failed treatment are not re-treated.
